# Image Segmentation Based on Relative Motion and Relative Disparity Cues in Topographically Organized Areas of Human Visual Cortex

**DOI:** 10.1038/s41598-019-45036-y

**Published:** 2019-06-26

**Authors:** Peter J. Kohler, Benoit R. Cottereau, Anthony M. Norcia

**Affiliations:** 1 Department of Psychology, Stanford University, Jordan Hall, Building 420, 450 Serra Mall, Stanford, CA 94305 USA; 2 Université de Toulouse, Centre de Recherche Cerveau et Cognition, Toulouse, France; 3 Centre National de la Recherche Scientifique, Toulouse Cedex, France

**Keywords:** Perception, Extrastriate cortex, Motion detection, Object vision

## Abstract

The borders between objects and their backgrounds create discontinuities in image feature maps that can be used to recover object shape. Here we used functional magnetic resonance imaging to identify cortical areas that encode two of the most important image segmentation cues: relative motion and relative disparity. Relative motion and disparity cues were isolated by defining a central 2-degree disk using random-dot kinematograms and stereograms, respectively. For motion, the disk elicited retinotopically organized activations starting in V1 and extending through V2 and V3. In the surrounding region, we observed phase-inverted activations indicative of suppression, extending out to at least 6 degrees of retinal eccentricity. For disparity, disk activations were only found in V3, while suppression was observed in all early visual areas. Outside of early visual cortex, several areas were sensitive to both types of cues, most notably LO1, LO2 and V3B, making them additional candidate areas for motion- and disparity-cue combination. Adding an orthogonal task at fixation did not diminish these effects, and in fact led to small but measurable disk activations in V1 and V2 for disparity. The overall pattern of extra-striate activations is consistent with recent three-stream models of cortical organization.

## Introduction

The boundaries between objects and their background give rise to discontinuities in multiple feature maps. The role of orientation cues in the early stages of figure-ground processing has been widely studied with both single-unit recordings and functional MRI^1–8^, but the contribution of two strongly related parallax cues – relative motion and relative disparity – has been less well studied. Relative motion and disparity cues each contribute independently to image segmentation and the perception of shape and the two cues can be combined to disambiguate 3D object and scene structure^9–11^. As for orientation cues, the processing of relative motion and disparity information requires a spatial comparison of local estimates. The degree to which spatial pooling occurs at the same cortical processing stage regardless of cue, and whether the visual system applies common computational mechanisms for different cues, remains unresolved. These questions are fundamental for understanding how cue extraction in early visual areas contribute to global scene segmentation and the early stages of object recognition.

Cells in V1 of the macaque are sensitive to local motion information and sensitivity to image discontinuities created by relative motion has been observed at both early and later visual processing stages. Single unit responses to relative motion information are robust in primate V1 and V2^12–17^, as well as in MT^18–21^ and IT^22^, although selectivity for the orientation of a motion-defined boundary may not arise until V2^17,23^. In humans, functional magnetic resonance imaging (fMRI) studies^24,25^ identified an area in dorsomedial occipital cortex, originally termed the kinetic occipital area (KO), that was activated by relative motion in texture-defined bars. However, other human fMRI studies at that time found that this stimulus also produces activations in V1, V2, V3 and in the hMT+ complex that includes the homologue of macaque MT^26–28^. Later work made more extensive measurements in topographically organized visual areas and used fMRI adaptation to identify selectivity for the orientation of motion boundaries in areas V3A, V3B, LO1, LO2 and V7^29^, which partially overlapped with functionally-defined area KO. Thus, while evidence for relative motion processing as early as V1 has been widely reported in the macaque single-unit literature, evidence from human fMRI is less compelling.

Studies in macaque suggest that image discontinuities generated by relative disparity are not encoded before V2^30–32^. Sensitivity to disparity discontinuities has also been found in V3^33^, V4^34,35^, IT^36^ and, depending on the precise definition of relative disparity, in MT^37^. In humans, the role of early visual areas V1-V3 in processing disparity discontinuities is again less well established. Several studies have compared fMRI BOLD responses to displays with multiple disparity planes to responses produced by a single depth-plane, and found dominant activations in V3A^38–40^. Because these studies contrasted non-zero disparity and zero disparity displays, the effects could be driven by tuning for absolute or relative disparity, or for both (see also^41^). This confound was addressed by Neri and colleagues^42^, who used fMRI adaptation to measure responses to both absolute and relative disparity separately. They found that areas V4 and V8 (and to a lesser degree early visual areas V1, V2 and V3) showed adaptation effects for both relative and absolute disparity, but that adaptation was only present for absolute disparity in V3A, MT and V7. A more recent study of cue combination reported reliable classification of depth-sign based on motion-defined accretion and deletion cues in V1, V2, and V3 as well as in a wide range of topographically organized areas outside early visual cortex. By contrast, disparity-defined depth sign could only be robustly decoded outside early visual cortex, especially in V3B/KO^9^.

Global figure-ground segmentation is often modeled as relying on a combination of border discontinuity and surface-based processes^43–46^. Local or “first-order” encoding of both motion and disparity cues can be modelled using similar energy-like computations executed within the classical receptive field of cells in V1^47,48^, but local computations are insufficient to define “second-order” features such as motion- or disparity-defined edges or surfaces. The specific nature of these representations and the stage of the human visual processing hierarchy at which they are first extracted, remains poorly understood. An fMRI study of relative motion processing^26^ suggested that local motion measurements are compared as early as V1, where BOLD activations were found to be concentrated at the retinotopic location of the border in figure-ground displays. No studies have examined whether disparity cues can drive a similar topographically specific representation. Disparity sensitivity, even at the local level, involves a prior combination of the inputs of the two eyes and thus relative disparity processing may require additional processing.

Whether early retinotopic visual areas support border- or surface-based representation of objects is differentiable in surface-based analyses of BOLD signals because border and surface-based representations will generate different retinotopically organized response profiles. We therefore compared the detailed topographic organization of relative motion and disparity responses in V1, V2 and V3. We also measured motion- and disparity-defined form sensitivity in 15 topographically organized visual areas outside early visual cortex. The latter analysis is motivated by previous results showing that figure-ground stimulus configurations such as the disk-annulus configuration are also expected to drive global shape processes and thus activate object-sensitive cortical areas^49–51^. Our disk-annulus stimulus configurations were presented using an experimental design that controls for the contribution of absolute motion and disparity signals to the measured responses. To probe the possible influence of attention-related feedback^1^ on motion- and disparity-defined form processing, we had participants either passively view the stimuli or perform an orthogonal task at fixation, in separate experiments. We find that motion and disparity-defined form processing was not influenced by the orthogonal task. Overall, areas that respond to relative motion also respond to relative disparity, but representations are more robust for motion than for disparity, especially in early visual areas. The raw data from Experiments 1 and 2 are available at http://openneuro.org/datasets/ds001984 and http://openneuro.org/datasets/ds001978, respectively. 

## Results

### Eccentricity analysis in early visual cortex

**Figure 1:**
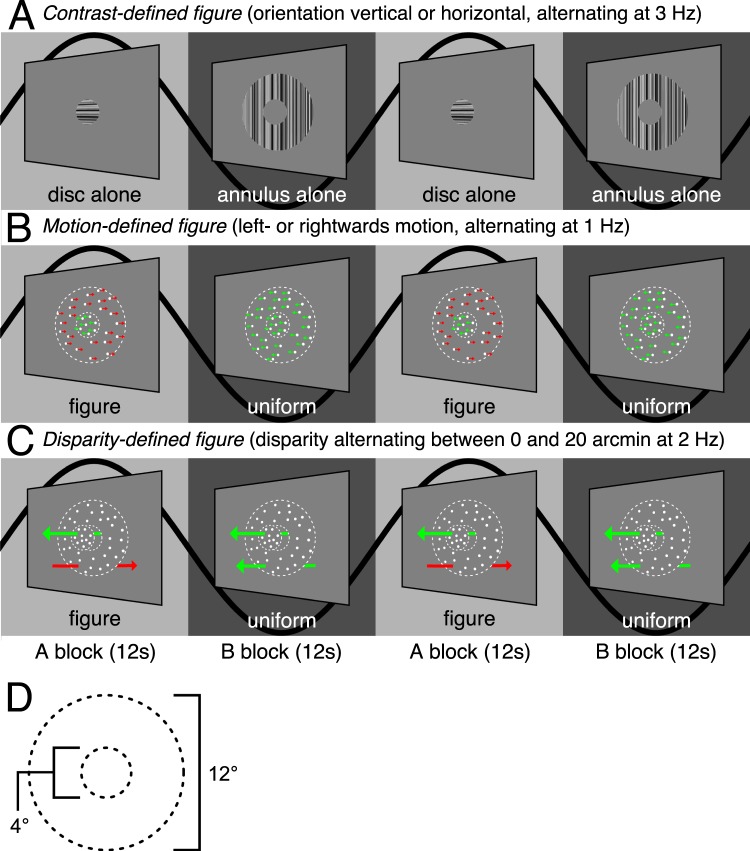
The experimental design used in Experiment 1. The on-off responses evoked by the conditions that are captured by our Fourier analysis, are illustrated with sine waves. (**A**) The contrast condition – the orientation of both the disk and annulus alternated between vertical and horizontal at 3 Hz. Disk and annulus were presented in temporal succession. (**B**) The motion condition – dots alternated between left- and right-wards motion at 1 Hz. When these direction changes were quadrature phase shifted between the disk and annulus regions, a figure percept was evoked, when alternations were in phase, there was no such percept. (**C**) The disparity condition – the dots alternated between the fixation plane (0 arcmin) and a position behind fixation (uncrossed disparity, 20 arcmin) at 2 Hz. Anti-phase movement of the disk and annulus regions led to a figure percept, while in phase movement generated no such percept. The white dotted line in B and C indicates the extent of the disk and annulus used in A. Note that the motion and disparity conditions were identical to those used in Experiment 2, except for the introduction of an RSVP task at fixation, which directed attention away from the stimulus. (**D**) Schematic outlining the size of the disk and annulus region, respectively, which was shared across all conditions.

**Figure 2:**
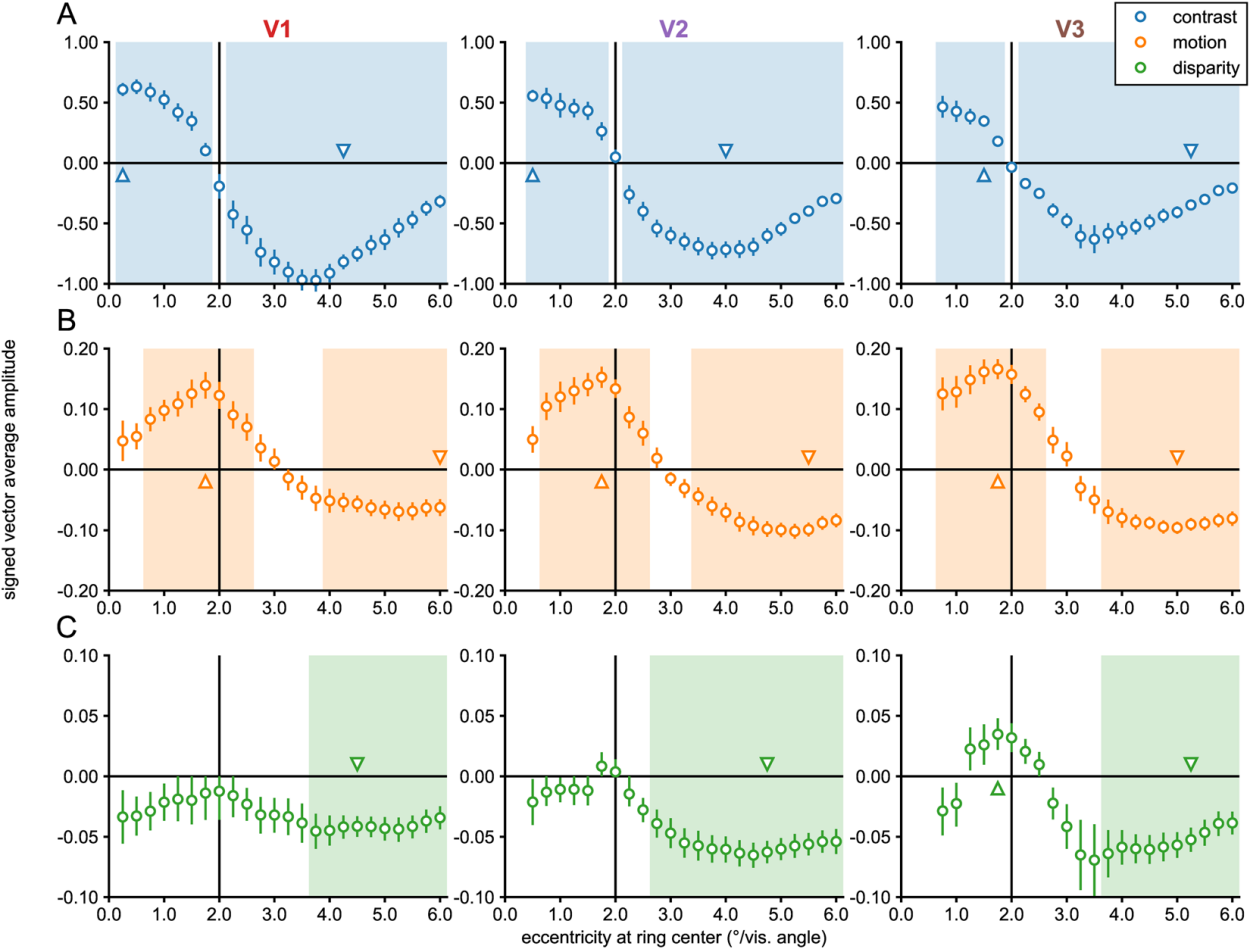
Eccentricity analysis of V1-V3 in Experiment 1. Signed vector-average amplitudes for the contrast (**A**), motion (**B**) and disparity (**C**) conditions within 24 sub-ROIs, defined based on a retinotopy template as centered on eccentricities spaced 0.25° apart, each having a width of 0.5°, as plotted on the x-axes. Note that V2 and V3 sub-ROIs centered at low eccentricities (0.25° for V2; 0.25° and 0.5° for V3) were not analyzed and plotted (see Methods). The shaded areas on the plots indicate condition × sub-ROI combinations that were significant at α = 0.05. The positively and negatively signed sub-ROIs that had the lowest *p-*values for each combination have been indicated with up and down-arrows, and single cycle averages have been plotted for these sub-ROIs in Fig. 3. For disparity, no V1 sub-ROIs were given a positive sign, and the two positively signed sub-ROIs in V2 were far from significance, so the positively signed cycle average for disparity was only plotted for V3, where the lowest positively signed *p*-value was close to significance (*p* = 0.06). The real and imaginary values used to compute the signed vector-average amplitudes have been plotted for the highlighted negatively and positively signed V3 sub-ROIs in Supplementary Fig. 1. The color of the text for the ROI names matches the ROI colors plotted on the inflated cortical surface reconstruction in Fig. 6.

**Figure 3:**
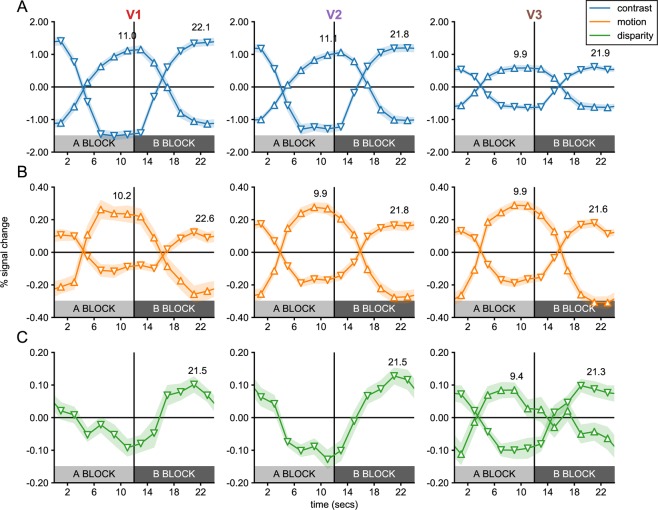
Single cycle averages for V1, V2 and V3 sub-ROIs in Experiment 1. Single cycle averages were computed from the most significant positively and negatively signed sub-ROIs in each visual cortex ROI following the color and marker convention of Fig. 2. The shaded region around each plot indicates the standard error of the mean, across participants. The delay of the peak response in seconds, estimated based on the phase, is plotted above each cycle average. Note that cycle averages have been plotted at the middle of each 2-second TR. The plot clearly demonstrates that positively-signed sub-ROIs (up-arrows) respond preferentially to the A block, while negatively signed ROIs respond preferentially to the B block (down-arrows).

We begin by describing results from early visual areas V1, V2 and V3, each of which have strong retinotopic representations of the visual field. To visualize and quantify the existence and retinotopic specificity of the representations of the motion and disparity-defined figures on the cortical surface, we used a retinotopy template^52^ to define eccentricity-based sub-ROIs and then used a fMRI localizer stimulus to verify the accuracy of the template. The goal of the sub-ROI analysis was to track the responses to central (figure surface), boundary and outer (ground) regions of the stimulus across the surfaces of the early visual areas. The localizer stimulus consisted of 12 sec blocks of high contrast dynamic textures presented in a central disk region, alternating with the same textures presented in an annulus surrounding the disk (see Fig. 1A). We estimated the response to the stimulus as the signed average amplitude values over the period of block alternations, derived by applying a Fourier transform to the ROI-average time course for each participant, computing the vector-average amplitude and phase by averaging the real and imaginary parts of the complex values at the stimulus frequency across participants, and then assigning a positive or negative sign to each amplitude based on the phase (see Methods for details). The real and imaginary values used to compute the vector-average amplitude and phase in Experiments 1 and 2 are plotted for two V3 sub-ROIs, as well as for V3B, in Supplementary Figs 1 and 2. We used a one-sample Hotelling’s *T*^*2*^-test to test if the real and imaginary parts of the complex values for each ROI were significantly different from [0,0]. The results are plotted for the contrast-based localizer condition in Fig. 2A. Positive amplitudes indicate relatively stronger responses to the A block, during which the disk was presented, compared to the B block, during which the annulus was presented. Negative amplitudes indicate the opposite. This can be observed in the single-cycle averages from the sub-ROIs that had the most significant positive and negative amplitudes in the localizer condition, plotted in Fig. 3A. For convenience, we will refer to the positive amplitudes in the localizer condition as “disk responses” and the negative amplitudes as “annulus responses”.

In areas of cortex defined by the template as responsive to eccentricities covered by the disk, we see high amplitude disk responses. At eccentricities defined as near the disk boundary (2° radius), the phase sign reverses and we begin to see annulus responses. There are significant responses at all eccentricities covered by the stimulus, except for the sign reversal region where voxels responding to the disk and the annulus, 180° out of phase, are likely mixed together. The pattern of results indicates that the template-based procedure allows us to accurately localize the eccentricity of the contrast-defined boundary in retinotopic cortex.

While the contrast condition alternated a disk and an annulus, the motion and disparity conditions alternated figure-ground and uniform field configurations (see Fig. 1). The dots updated dynamically at 20 Hz throughout each block, and in the motion condition, they were temporally correlated with a long lifetime. In the disparity condition, they were temporally uncorrelated across updates, and thus did not generate monocular cues to form. In the motion condition, dots alternated between left- and rightwards movement at 1 Hz, while in the disparity condition they alternated between the fixation plane (0 arcmin), and a position behind fixation (uncrossed disparity, 20 arcmin) at 2 Hz. For both conditions, the disk and annulus region alternated in anti-phase during the A block, and in phase during the B block. Importantly, absolute motion and absolute disparity were alternating during both A and B blocks, but only the A block generated relative motion and disparity cues that give rise to a figure boundary. The design and corresponding Fourier analysis yields the differential response between the blocks and thus allows us to isolate responses driven by the presence of relative motion/disparity discontinuity, the segmented figure surface or some combination of the two, from responses driven by absolute motion and disparity.

The overall response amplitudes in the motion and disparity conditions were 5–10 times weaker than in the contrast condition, but there were nonetheless significant activations at multiple eccentricities. For both motion and disparity, positively signed amplitudes indicate stronger responses to the A block in which the figure and surround were segmented, while negatively signed amplitudes indicate stronger responses to the B block in which a uniform field was perceived. As we discuss the results, we will use the terms “figure responses” and “uniform responses” to refer to positively and negatively signed amplitudes, respectively. Note that these terms are meant to strictly refer to the phase sign of the response relative to the A and B blocks, and not as an interpretation of the data. As noted in the Introduction, figure responses can be driven by enhancement of the figure boundary or surface, or by a mixture of these two forms of enhancement. Uniform response indicate that when the figure is present, responses are weaker than when the figure is not present. Uniform responses that are measured in the region outside the figure are likely due to suppression of the surround when the figure is present, rather than surround-exclusive enhancement of responses when the field is uniform.

The motion condition (see Fig. 2B), produced figure responses at the disk boundary in all early visual areas, which persisted inside the disk region and could also be observed ~1° outside the disk. Surprisingly, we also saw uniform responses at eccentricities beginning ~3°, which were significant in all visual areas. In the disparity condition (see Fig. 2C), we did not measure significant figure responses in V1 or V2, and in fact hardly ever observed positively signed amplitudes, according to our phase-derived definition (see Methods). No V1 sub-ROIs had positive amplitudes, and the only two V2 sub-ROIs that did, were far from significant (*p*’s > 0.7). By contrast, a region of six V3 sub-ROIs centered on the boundary and extending ~0.5° inside and outside the boundary were given positive signs. The two sub-ROIs at and immediately inside the boundary approached significance (*p*’s < 0.08; smallest *p* = 0.06). We also observed significant uniform responses in all early visual areas. Single cycle averages for the figure and uniform responses are plotted for motion and disparity in Fig. 3B,C. Figure responses had similar shapes and estimated delays to the disk responses in the localizer conditions, while uniform responses in both conditions were similar to the annulus responses.

These results suggest an interesting dichotomy: Among the early visual areas, we only see figure responses for relative disparity in V3, while all three early visual areas have uniform responses. As mentioned above, the uniform responses measured for motion and disparity are likely the result of suppression when figure is present. This relative suppression of surround-region responses may be due to feedback from higher cortical areas, driven by top-down attentional selection. Spatial attention can modulate BOLD responses in all early visual areas, including V1^53–55^. We directly tested the hypothesis that the suppression is due to attention-driven feedback by running a second experiment.

**Figure 4:**
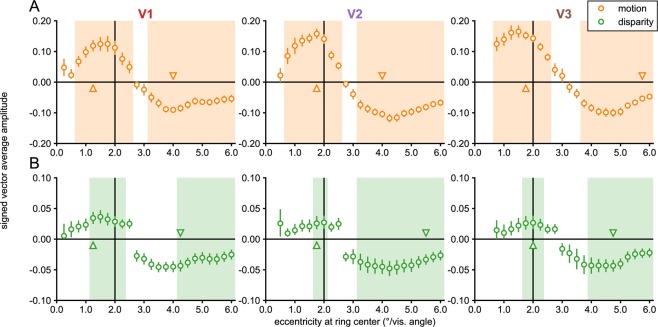
Eccentricity analysis of V1-V3 in Experiment 2. Signed vector-average amplitudes within 24 eccentricity-defined sub-ROIs, centered on eccentricities spaced 0.25° apart, each having a width of 0.5°. The shaded areas on the plots indicate condition × sub-ROI combinations that were significant at α = 0.05. Note that V2 and V3 sub-ROIs centered at low eccentricities (0.25° for V2; 0.25° and 0.5° for V3) were not analyzed and plotted (see Methods). The up and down-arrows respectively indicate the positively and negatively signed sub-ROIs that had the lowest *p-*values for each combination. The real and imaginary values used to compute the signed vector-average amplitudes have been plotted for the highlighted negatively and positively signed V3 sub-ROIs in Supplementary Fig. 2. The color of the text for the ROI names matches the ROI colors plotted on the inflated cortical surface reconstruction in Fig. 6. The motion and disparity conditions plotted here were identical to the ones used in Experiment 1 (see Fig. 2), except attention was directed away from the stimulus.

**Figure 5:**
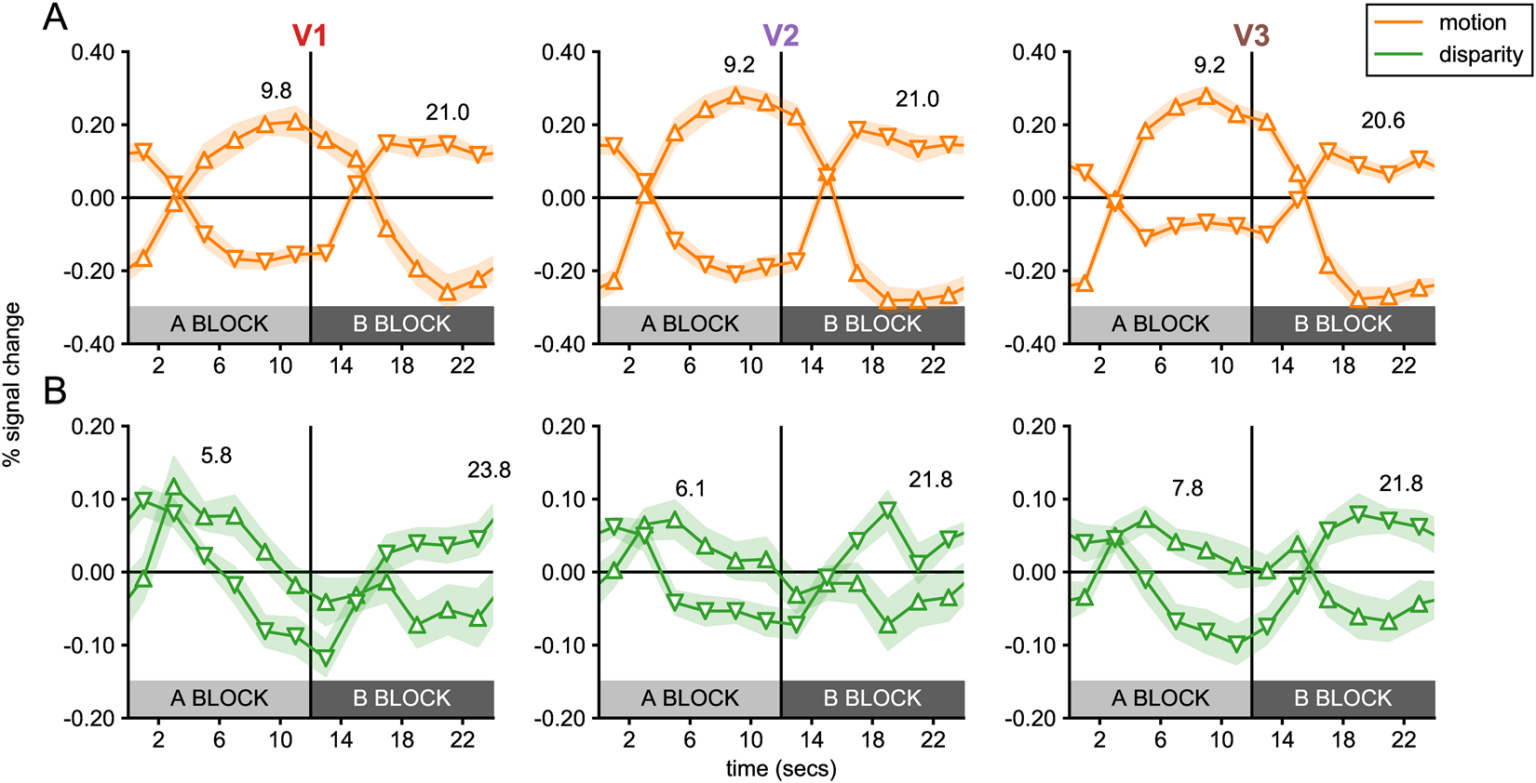
Single cycle averages for V1, V2 and V3 sub-ROIs in Experiment 2. Single cycle averages computed for the most significant positively and negatively signed sub-ROIs in each visual cortex ROI, following the color and marker convention of Fig. 2. The shaded region around each plot indicates the standard error of the mean, across participants. The delay of the peak response, estimated based on the phase, is plotted above each cycle average. Note that cycle averages have been plotted at the middle of each 2-second TR. The plot clearly demonstrates that positively-signed sub-ROIs (up-arrows) respond preferentially to the A block, while negatively signed ROIs respond preferentially to the B block (down-arrows).

Experiment 2 used the same parameters as the motion and disparity conditions in Experiment 1, except that attention was now directed away from the stimulus via an orthogonal task presented at fixation (see the ‘*Visual stimuli’* section in the Methods). Response profiles were generally very similar across the two experiments. We tested this by performing independent samples Hotelling’s *T*^*2*^-tests comparing the data between the two experiments for each of the 69 sub-ROIs across the three visual areas (see Methods). None were significant for the disparity condition and only 3 for the motion condition, both in the V1 periphery where amplitudes were slightly larger for the passive viewing condition (in sub-ROIs centered on 5.5°, 5.75° and 6.0°; biggest *p* = 0.005). As expected from the failure to detect consistent significant differences between the experiments, both motion and disparity produced uniform responses that were largely similar between the experiments. Yet, despite the results of the between-group test, the disparity condition in Experiment 2 produced significant figure responses, as measured with a one-sample test against 0, near the figure boundary in all three early visual areas (see Fig. 4), in contrast with Experiment 1 where evidence of disparity-driven figure responses was only seen in V3 (see Fig. 2). Single-cycle averages were also quite similar across the two experiments, although the delays of the peak figure response were perhaps slightly shorter for disparity in Experiment 2, compared to the motion condition and the other conditions in Experiment 1 (compare Figs 3 and 5).

These results lead us to reject our hypothesis that the uniform responses we saw in the surround region in Experiment 1 are due to top-down attention-mediated suppression of the surround. Instead, the results if anything suggest that spatial attention elicited by the orthogonal task at fixation may in fact enhance disparity-driven figure responses in V1 and V2, making weak signals measurable. In V3, figure responses are present in both experiments, but the figure responses are perhaps more robust with the orthogonal task (where 3 sub-ROIs reached significance).

### Extended ROI-based analysis of responsivity

The next set of analyses quantifies motion and disparity sensitivity in topographically organized visual areas beyond early visual cortex. Here the signed vector-average amplitude was computed based on the average time course across all voxels within each ROI. As for the sub-ROIs in early visual cortex, positive amplitudes indicate responses to the figure (A block), while negative amplitudes indicate stronger responses to the uniform field (B block).

**Figure 6:**
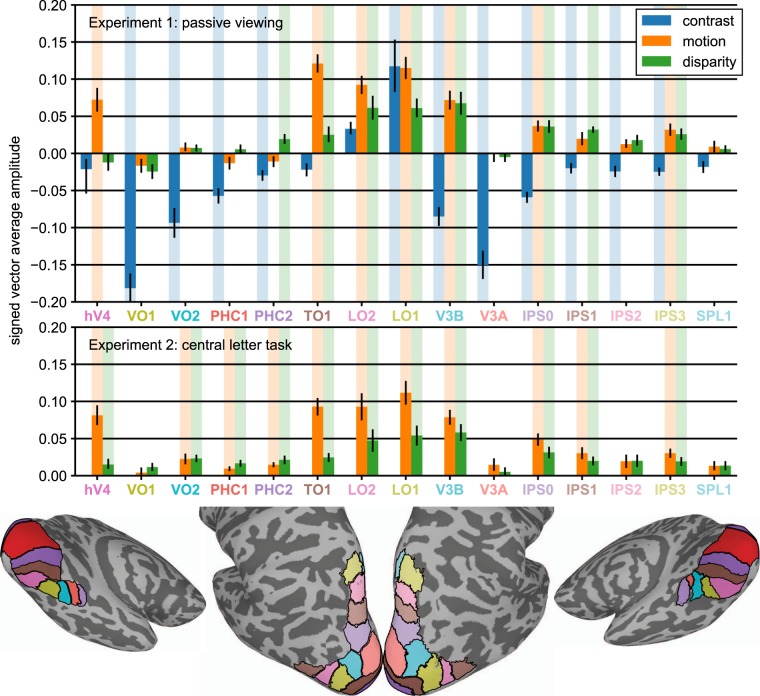
ROI results from Experiments 1 and 2. Signed vector-average amplitude within 15 topographically organized regions of interest, excluding early visual areas V1-V3, which are plotted in Figs 2–5. The contrast condition is shown in blue, the motion condition in orange, and the disparity condition in green. Shaded areas behind the bars indicate condition × ROI combinations that were significant at α = 0.05. The ROIs are shown for the both hemispheres of an example participant’s inflated cortical surface reconstruction below the graph.

In both the motion- and disparity-defined form conditions, all significant activations (*p* < 0.05; indicated with shaded areas in Fig. 6) were positively signed. This is consistent either with positive-sign activations generated by the figure overcoming any negatively signed activations that may have occurred in a subset of voxels within a given ROI, or a lack of negatively signed activation. We distinguished three response patterns: areas that only had significant responses to the motion-defined figure (hV4), areas that only had significant responses to the disparity-defined figure (PHC2, IPS1) and areas that had significant responses to both (TO1, V3B, LO1, LO2, IPS0, IPS3). Our results suggest a clear functional distinction between V3B and nearby area V3A, which has no significant responses. Motion generated stronger responses than disparity in V3 (see Figs 2 and 4), but this was less pronounced among the higher-level areas: Both V3B, IPS0 and IPS3 had nearly identical responses to the two conditions.

For comparison, we also plot the contrast-based localizer condition in Fig. 6. Most ROIs produce negatively signed responses, indicating that more voxels are responding to the annulus compared to the disk. LO1 and LO2 are the only exceptions to this rule, and the positively signed responses (which reach significance in LO1) are likely a result of the known preference of these areas for global shapes^49^. It is also worth noting that several ROIs that produce weak, non-significant responses in the motion and disparity conditions, nonetheless respond strongly in the contrast condition (VO1, VO2, PHC1, V3A), indicating that the observed variability in motion and disparity sensitivity cannot be explained by reference to general difficulties in measuring responses from specific ROIs.

### Effect of attention on disparity-defined figure activations outside of early visual cortex

The results of Experiment 2 are shown in the bottom half of Fig. 6. Did the orthogonal task influence the responses to motion and disparity? Experiment 2 produced significant positively signed responses to motion, as measured with a one-sample test against 0, in every area that had reached significance in Experiment 1, as well as in VO2, PHC1, PHC2 and IPS1, although the independent samples test comparing the motion conditions across the two experiments only reached significance in PHC2 (*p* = 0.044; all other *p*’s > 0.15). For disparity, we again replicated every significant effect from Experiment 1, with additional significant effects observed in hV4, VO2, and PHC1. The independent samples test reached significance in VO1 (*p* = 0.029), an area for which none of the single sample tests reached significance, and approached significance in VO2 (*p* = 0.065; all other *p*’s > 0.25). These results suggest that the figure responses we measured for motion- and disparity are mostly independent of attention, at least for the tasks we used here. The small effects that we do observe, mostly in ventral areas, are in the form of enhanced figure responses, similar to what we observed for disparity in V1 and V2 sub-ROIs.

### Whole brain analysis

**Figure 7:**
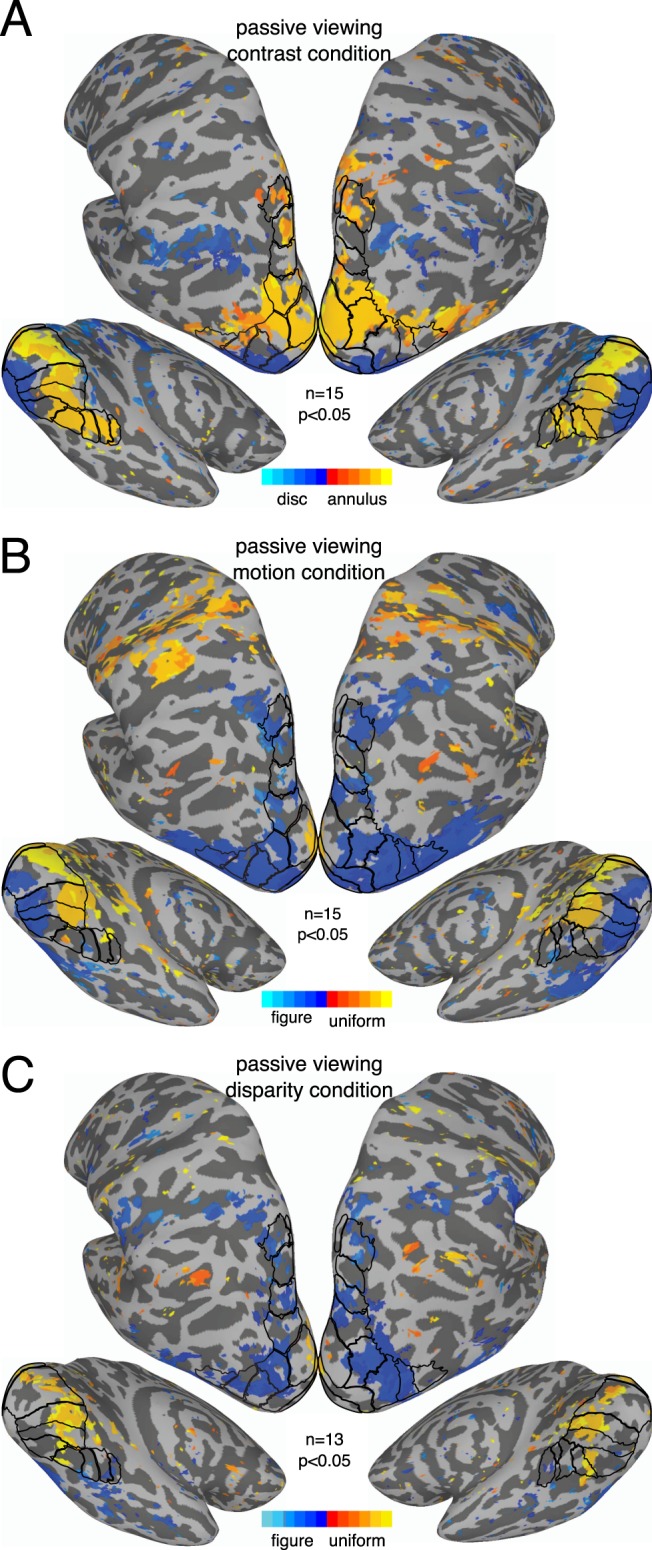
Whole-brain results from Experiment 1. Vector-averaged phase maps, converted to seconds of delay, thresholded at α = 0.05. Maps were produced through surface-based alignment procedure in which each subjects’ cortical mesh was converted to a standardized mesh, which allowed for cross-subject comparisons of values at each mesh node. The vector-average phase across subjects, as well as a corresponding *p*-value based on both amplitude and phase, could then be computed for each mesh node. As indicated by the color bar, orange shades indicate responses to the A block, while blue shades indicate responses to the B block. The ROIs are outlined on the surface, and labeled versions can be inspected in Fig. 6.

A surface-based alignment approach was used to visualize vector-averaged responses to the conditions in the two experiments, across all of cortex, including regions outside our set of ROIs. The results of these analyses did not deviate from the sub-ROI and whole-ROI analyses in any material way, and we will only describe them briefly. We plot the phase of the vector-averaged response, converted to seconds of delay, and thresholded by significance. Blue colors indicate responses to the A block, while orange colors indicate responses to the B block. For the contrast condition (see Fig. 7A), the reversal of the phase sign from disk responses to annulus responses between low and high eccentricities, described in the sub-ROI analysis (see Fig. 2A) can be clearly observed in early visual areas.

**Figure 8:**
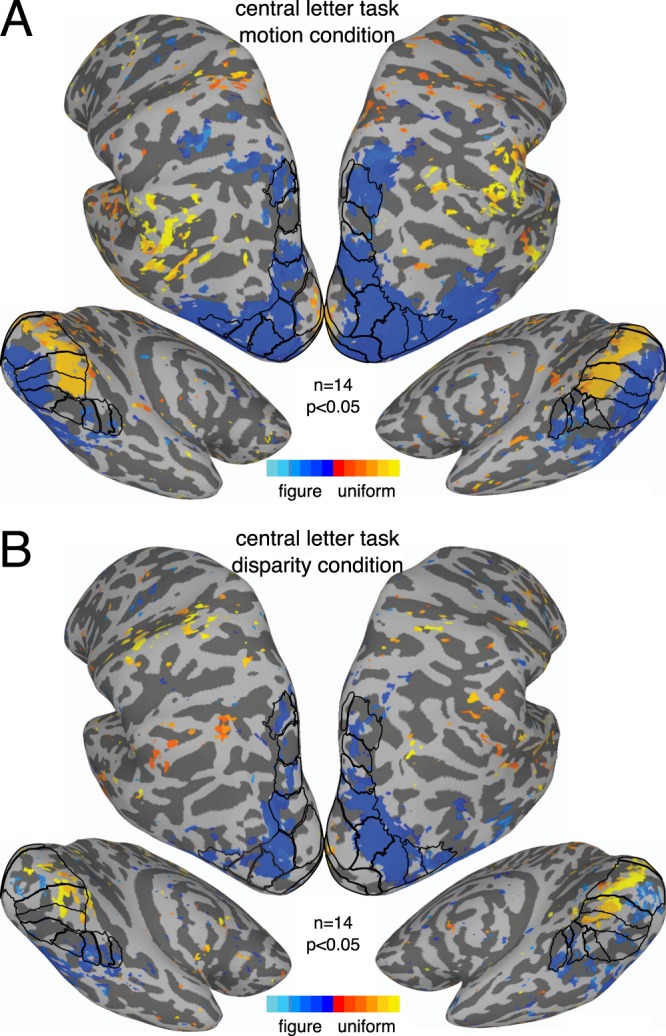
Whole-brain results from Experiment 2. Vector-averaged phase maps, converted to seconds of delay, thresholded at α = 0.05. Maps were produced as described for Fig. 7 and in the text. As indicated by the color bar, orange shades indicate responses to the A block, while blue shades indicate responses to the B block. The ROIs are outlined on the surface, and labeled versions can be inspected in Fig. 6.

For motion, we see clear evidence of the figure region response in early visual cortex near the occipital pole (Fig. 7B, blue colors) and uniform responses consistent with suppression of the surround more anteriorly (orange colors). We also see figure responses that cover most dorsolateral ROIs and extend anteriorly and ventrally beyond the ROIs. The figure response is weak or absent in V3A or IPS2, but strong in nearby areas such as V3B, LO1, IPS0 and IPS3. The general pattern is similar in Experiment 2 (see Fig. 8A), although figure responses in ventral areas are perhaps slightly more pronounced.

For disparity, we again see uniform responses consistent with suppression in early visual cortex (see Fig. 7C, orange colors). It is worth noting that for both motion and disparity, in both experiments, there is little evidence of suppression outside of early visual cortex, at least within our ROIs. Figure responses can be observed in early visual cortex in Experiment 2 (see Fig. 8), although they are fairly spotty and likely benefitted from the increased sensitivity of the sub-ROI analysis (shown in Figs 2 and 4). Both experiments produce figure responses in V3B, LO1, and several IPS areas, but not in V3A. As for motion, figure responses in ventral areas are perhaps slightly stronger in Experiment 2, than in Experiment 1.

## Discussion

Figure-ground segmentation could be supported by several mechanisms that may act separately or in combination, including, as noted by Likova and Tyler^56^, retinotopic enhancement of the figure boundary, retinotopic enhancement of the figure surface and retinotopic suppression of the ground region. Our detailed measurements of the representation of motion- and disparity-defined forms in strongly retinotopic visual areas V1, V2 and V3 provide evidence that all three of these mechanisms are implemented in early visual cortex, at least for motion. Our measurements in retinotopic areas outside early visual cortex demonstrate that with a few important exceptions, areas that respond to one cue also respond to the other. Responses to both cues are stronger in lateral and dorsal visual areas compared to areas on the ventral surface of cortex, extending a similar pattern found in previous studies to a wider range of areas.

### Representations of the figure boundary and surface in early visual areas

Our results provide clear evidence for enhancement of the figure boundary. For motion, we find measurable figure responses around the boundary in all early visual areas and in both experiments. For disparity, figure responses near the boundary are measurable in V3 in Experiment 1, and in all early visual areas in Experiment 2.

The evidence for surface enhancement is more ambiguous. For the motion and disparity conditions, figure responses are weaker towards the center of the figure and become non-significant at low eccentricities for V1 and V2. However, the drop off is more pronounced on the outer side of the figure boundary than on the inner side. This is especially clear for the motion condition where responses are strong across all early visual ROIs in both experiments. This asymmetry indicates that voxels with receptive fields centered inside the figure boundary are more likely to produce a figure response than those just outside the boundary. Are voxels inside the figure simply responding to the figure boundary, rather than the surface? Population receptive fields measured in voxels increase in size towards the periphery^57^, so voxels outside the boundary are more likely to have receptive fields that overlap with the boundary, compared to those inside. This means that boundary-exclusive responses should be more pronounced outside the figure, compared to inside, the opposite of the asymmetry we observe. So, while responses near the center of the figure become weaker compared to the contrast condition, there is clear evidence for some form of surface enhancement for motion-defined form. Disparity may also give rise to surface enhancement, but this is harder to measure because early visual cortex responses are generally less robust for disparity than for motion.

It is perhaps surprising that we see any figure responses outside the figure boundary. This likely reflects two factors, one is the size of the receptive fields that best respond to motion and disparity and the other the relative balance of more global processes that leads to retinotopically organized enhancement and suppression. The apparent size of the center and surround regions may depend on both these factors. If the underlying receptive fields are relatively large and the strength of enhancement is greater than that of suppression, then the disk representation may appear to be expanded.

Phase-inverted responses consistent with suppression of the ground region (see below) were measured across all early visual areas for both motion and disparity and this suppression is independent of the task we used. Thus, for motion-defined form we have strong evidence for boundary and figure enhancement as well as ground suppression in all three early visual areas. For disparity, we have strong evidence for boundary enhancement and ground suppression for disparity, but no conclusive evidence of figure enhancement.

### Basis of opposing sign responses

The patterns of activation we observed in V1, V2 and V3 for motion- and disparity-defined figures each consisted of a region of positively signed activation surrounded by a negatively signed activation. The negatively signed activation in the surround could arise from suppression of responses during the A block or enhanced responses during the B block. We consider the former more plausible for several reasons. First, the center and surround region are indistinguishable in the uniform stimuli used in the B blocks, and it is thus unlikely that there would be selective enhancement of the surround. Second, an opposing sign organization like the one we have observed has been found previously in macaque for “first-order” contrast-defined stimuli when comparing blank screen baseline activation levels to those within and adjacent to the retinotopic location of high contrast patterns^58–60^. Single unit and local field potential recordings in the retinotopic and adjacent representations of spatially localized, high contrast stimuli indicate suppression of baseline neural responses in regions that had negatively signed BOLD^58^. Our results suggest that similar suppression is occurring with our motion- and disparity-defined “second-order” contrast stimuli. Suppression of spiking activity from the ground region by the presence of a figure has been reported for orientation-defined textures in macaque V1^2^.

The combination of figure/surface enhancement and surround suppression may thus be a common computational strategy across several cue types, including contrast^58,59^, texture^2^, as well as the motion and disparity cues measured here. The strength of the spatial antagonism of responses in early visual areas may differ across cues, perhaps because disparity requires binocular processing, while other cues can be processed monocularly. Nonetheless, our results, in combination with existing results in the literature, suggest that there is a substantial degree of invariance in the computational strategy applied for motion, disparity, contrast and texture as early as V1. Interestingly, in our hands both enhancement and suppression are largely independent of task.

### Comparison with previous fMRI studies of figure/ground segmentation

The center-surround configuration of our disk stimuli lends itself to the detection of alternate-sign activations because eccentricity is mapped systematically on the cortical surface in the foveal confluence-region of early visual cortex^61^. In the motion-domain, Reppas and colleagues^26^ observed positive activation at the border of a disk-shaped motion-defined form, but found neither negative activation in the ground region nor positive activation within the figure region, as we see in our data. This difference may be due to an ice-berg effect in the older dataset, where only the strongest activations rise above the noise floor. A suggestion of negative BOLD activation was present in the disparity data of Parker and Bridge^62^, but their use of rotating wedge-shaped stimuli complicated its visualization and measurement. A study in which a second-order figure region (a bar) was defined by temporal transients^56^ did find negative activation adjacent to the retinotopic locus of the bar, but that study did not find enhanced activation within the retinotopic representation of the figure as we did. Texture-defined figures have also been found to increase overall activation in early areas^5^, but the analysis did not consider figure, boundary and ground regions separately as we have done here. Finally, isolated background suppression has also been found for luminance-defined shapes, but this study did not evaluate figure enhancement effects^63^.

### Role of top-down influences

Several studies have suggested that figure/ground representations in V1 depend on influences from higher-order visual areas. For example, figure enhancement for texture-defined form that is measurable in V1 in awake animals is reduced by anesthesia^64^. Simultaneous recordings in V1 and V4 suggest that surround-region suppression in V1 for texture-defined forms is controlled by an input from V4^1,2^. These previous studies motivated our use of two different behavioral tasks in our study of motion- and disparity-defined form. In one task, the participants were instructed to fixate in the center of the figure region and to pay attention to the stimuli, in the other they were given a difficult letter discrimination task to perform in which the letters overlapped the figure region. The most prominent effect of the task was that responses to the disparity-defined figure boundary became measurable in V1 and V2 when participants were performing the letter task. Although the direct comparison of Experiments 1 and 2 did not reach significance in any sub-ROIs near the figure boundary, this difference may reflect a true effect of attention. Such an effect would indicate that allocating spatial attention to a region of the visual field can upregulate weak figure processing in early visual cortex, in addition to the stimulus-independent modulation of responses previously observed^53–55^. This is an intriguing possibility that should be explored further.

The task had limited influence on negatively signed activations in early visual areas. It is thus possible that the suppression we observe may depend on feedback mechanisms that are relatively independent of task-related deployment of attention. Early visual areas receive direct feedback connections from a wide range of cortical areas, including superior and inferior temporal cortex^65^, V2 and MT (see^66^ for review).

### Comparison with single-unit studies of boundary and figure responses in EVC

Single-cell recordings have identified selectivity for motion-defined edges as early as V1^13,15^, but similar selectivity for disparity has been observed only as early as V2^67^. The extraction of spatial cue-relationships necessary for detecting an edge could be made over relatively small distances, while surround modulation requires longer-range spatial interactions. We find suppressed responses over the full extent of our stimulus presentation, so as far as 6° of radius and 4° from the border of the figure region. Several single-unit studies of texture segregation have explored the spatial organization of responsiveness to second-order figure-ground displays by placing the receptive field (RF) of V1 neurons at different locations within the figure, at its border or on the background region. These prior results from texture- and motion-defined form demonstrate enhanced responses to the figure and boundary region, relative to responses when the receptive field was on the background^1,2,64,68^, broadly consistent with our results.

Our fMRI block-design contrasted responses during periods where the field was segmented (A blocks) and periods where it was uniform (B blocks). To observe both figure enhancement and suppression of the background in the presence of a figure, it is critical to compare responses to segmented and uniform fields, and measure responses in both the figure and surround regions. Most single-unit studies have not done both. The single study of disparity-defined form found figure region enhancement in V1 measured relative to a uniform field, but no measurements were made in the surround^69^. The only single-unit study that has done both highlights the necessity of contrasting segmented and uniform field responses: Poort and colleagues showed explicit enhancement of activity in the figure region and suppression of the background, using texture-defined figures^2^. The measured suppression was sufficiently long-range that a figure presented in one hemisphere could elicit suppression in the other^2^. This is consistent with the suppression we measure at locations several degrees beyond the figure/ground border.

### Figure/ground representation in higher-order visual areas

We observed stronger responses to relative motion compared to relative disparity in early visual areas (see Fig. 2). This difference was present, but less pronounced in higher-level areas with responses being especially similar in V3B, IPS0 and IPS3, as has also been observed by Bridge and Parker^62^. Our results are also consistent with the ‘single-cue’ classification accuracies reported by Ban and colleagues, which are greater for motion than disparity in early visual areas, but more comparable in higher-level areas^9^. In V3A, their ‘single cue’ classification accuracies are above-chance for stimuli that do not produce significant responses in our data. The classifier may be picking up tuning for absolute motion and disparity^9^, which was controlled for in our experiment design. In this interpretation, V3A is sensitive to absolute, but not relative, motion and disparity, while neighboring V3B is sensitive to relative motion and disparity.

Outside of early visual cortex, we found that all areas that respond to one cue respond to the other cue in at least one of the two experiments. Areas TO1, LO1, LO2, V3B, IPS0 and IPS3 were jointly responsive in both experiments, while hV4, VO2, PHC1, PHC2 and IPS1 were jointly responsive only in Experiment 2. For the latter group of ROIs, joint responsiveness thus depends on attentional enhancement of weak processing of figures defined by one of the two cues, likely driven by a figure-specific effect of spatial attention, as described above for early visual cortex. Areas that are jointly responsive to the two cues are candidate regions for cue-combination for motion and disparity information. The task-independent responsiveness found in TO1, LO1, LO2, V3B, IPS0 and IPS3 may provide a more robust foundation for combining the two cues. Our analysis does not specifically address whether cue-combination occurs in the areas we find to be responsive to both cues, but it does provide a set of candidate areas in which cue combination could possibly occur. Ban and colleagues^9^ found evidence of integration of motion and disparity in V3B/KO, which partially overlaps with several of the areas we find to be sensitive to both cues: V3B, LO1, LO2 and V7/IPS0. This does however leave several jointly responsive areas that do not overlap with V3B/KO: TO1, IPS1, IPS3, hV4, VO2, PHC1 and PHC2, of which TO1 and IPS3 were jointly responsive in both experiments.

### Possible limitations

As suggested by prior single-unit work, the fundamental contrast to be made is between segmented and uniform fields. Our fMRI block design allowed us to measure this contrast and compare responses during segmented A blocks and uniform B blocks. However, for the motion conditions, the A block alternated between uniform and segmented fields, while in the disparity A block the field was always segmented. This difference is unlikely to have changed the spatial pattern observed in early visual areas, but could affect the relative magnitude of motion and disparity responses across visual cortex. Resolving this second-order effect requires further study.

### Three streams

Relative disparity processing has been associated with the “canonical macaque ventral stream” leading from V4 to IT^70–72^. We find that hV4 is responsive to both disparity and motion cues (although only in Experiment 2), which is consistent with reports that macaque V4 is sensitive to both relative disparity^34,35,73^, and relative motion^74^. In the macaque ventral stream, areas down-stream of V4 include sub-divisions of IT, TEO and TE, that are responsive to relative disparity^75,76^ and to relative motion^22,77^. In our data, responses to both motion- and disparity-defined form are weak or unmeasurable in ventral areas downstream of hV4 (VO1, VO2, PHC1, PHC2), but strong in areas that lie on the lateral surface (LO1, LO2 and V3B). This difference in responsiveness is not likely due to instrumental factors as the contrast-based localizer condition produces robust responses in at least three of the ventral areas (VO1, VO2 and PHC1). Thus, for motion- and disparity-defined forms, the functional homology is poor between human ventral-surface areas and the canonical macaque ventral areas, but better between human lateral areas and macaque ventral areas. Two recent proposals divide human visual cortex into three streams (dorsal, ventral and lateral), rather than the two canonical dorsal and ventral streams of the macaque^78,79^. Our data suggests that a functional homology exists between human lateral areas and the macaque ventral areas in IT cortex. Human ventral surface areas may either lack clear homologues in the macaque, or the homologous areas remain to be discovered.

## Methods

### Participants

15 healthy adult participants (5 female; mean age = 30.6 ± 13.5) participated in Experiment 1 and 14 participated in Experiment 2 (7 female; mean age = 31.9 ± 14.2), with 8 participants taking part in both experiments. Each participant had visual acuity better than +0.1 LogMar (20/25) in each eye as measured on a Bailey-Lovie chart and stereo-acuity of 40 seconds of arc or better on the RandDot stereoacuity test. The experiment began after the procedures of the study had been explained and the participant had given written informed consent. Experiment protocol and consent forms were approved by the Stanford University Institutional Review Board, and all methods were performed in accordance with the relevant guidelines and regulations.

### Visual stimuli

The stimuli for both experiments were shown on a 47” Resonance Technology LCD display and viewed through a mirror at a distance of 277 cm. This resulted in a presentation area of 12.1 × 21.2°/visual angle, of which our stimuli occupied 12 × 12°. The screen resolution was 1024 × 768 pixels, 8-bit color depth and a refresh rate of 60 Hz. In the relative disparity condition, the mean luminance was 2.17 cd/m^2^ and contrast was 60%, and stereoscopic stimuli were displayed using red/blue anaglyph glasses, which were worn throughout the experiment. In the other two conditions, the mean luminance was 34.49 cd/m^2^ and contrast was 90%.

For each stimulus condition, the display comprised a central 2° radius disk region and an immediately adjacent 6° radius annulus. In the relative motion condition, the disk-boundary was defined using a random-dot kinematogram. In the relative disparity condition, the disk and the annulus were defined using dynamic random-dot stereograms with no monocular cues. For the relative disparity display, dot size was 5 minutes of arc (arcmin) and dot density was 36 per (°/visual angle)^2^, while in the relative motion display, dot size was 10.4 arcmin and dot density was 10 per (°/visual angle)^2^. In Experiment 1, we also ran a boundary localizer condition in which the disk-annulus boundary was defined by a contrast difference in texture patterns comprised of 1-dimensional noise which alternated between horizontal and vertical orientations at 3 Hz. This condition allowed us to compare the boundary activations found in the motion and disparity conditions to the activations generated by a contrast-defined boundary to which all visual areas should be highly sensitive. This localizer also served to verify the accuracy of the retinotopy template^52^ we used.

In the contrast condition, the central disk was presented in what we will refer to as the “A block” of the fMRI design and alternated with the adjacent annulus configuration, presented during the “B block” (see Fig. 1A). In the relative motion condition, the horizontal positions of individual dots comprising a random dot pattern updated at 20 Hz. Dots were displaced by 10 arcmin per update (3.33°/sec) with a dot life-time of 100 video frames. The dots moved leftwards or rightwards, changing direction at 1 Hz. When these direction changes are quadrature phase shifted between the inside and the outside of the central disk, the dots move in opposite directions during half of each cycle, and in the same direction during the other half. When changes occur in phase, dots move in the same direction throughout the cycle. Motion in opposite directions produces a relative motion defined spatially segmented percept with a visible boundary between the disk and annulus regions. Motion in the same direction produces uniform motion with no boundary. In the A block, direction changes were made to occur out of phase between the center and surround, producing square-wave alternations between uniform motion and segmented configurations at 1 Hz. In the B block, direction changes were made to occur in phase. Locally, each part of the display contained dots that alternated between leftward and rightward motion at 1 Hz, only the relative direction of motion over the disk and annulus regions differed between A and B blocks.

In the relative disparity condition, the positions of individual dots updated at 20 Hz such that the dot fields were binocularly correlated, but temporally uncorrelated (no monocular cues). The horizontal disparity of the central disk and the annulus alternated at 2 Hz between 0 disparity and 20 arcmin of uncrossed disparity. In the A block, the disk and annulus alternated in anti-phase, generating a spatially segmented percept with a visible boundary between the disk and annulus regions defined by relative disparity. In the B block, the disk and annulus alternated in phase, leading to a uniform motion percept with no border. Thus, disparity modulated between 0 and 20 arcmin at all locations in both A and B blocks, with only the relative disparity over the disk and annulus regions differing between A and B blocks. Participants wore anaglyph glasses throughout the experiment, but the contrast and motion condition were identical in both eyes and thus effectively shown at 0 disparity.

In Experiment 2 we replicated the relative motion and disparity conditions from Experiment 1, but introduced a rapid serial visual presentation (RSVP) task at fixation that served to direct attention away from the stimulus. Subjects attended to an array of five letters, presented in trials of three array updates. Each trial consisted of a pre-target array of five randomly oriented Fs, followed by a target array, followed by a masker array identical to the pre-target array. The target array consisted of five Ls in the same positions and orientations as the pre-target Fs, but on a fraction of trials a randomly selected L was replaced by a T. On each change, subjects had to indicate with a button-press whether the target array was a T among Ls, or all Ls. The array duration was adapted online using a staircase procedure to stabilize performance at a constant level (~80% correct) during both A and B blocks. The letter display was superimposed on the motion and disparity stimuli, covering the central part of the disk without overlapping with the boundary.

### fMRI experimental procedure

We used a block design in which 12 s A blocks alternated periodically with 12 s B blocks, yielding a 24 s base period for the paradigm that was repeated 10 times in what we refer to as a “scan”. The design is illustrated schematically in Fig. 1. Ten stimulus cycles were shown per scan, with an additional half-cycle (one 12 s control block) being shown in the beginning of the scan to allow the brain and the scanner to settle. The data collected during this “dummy” period were removed from the fMRI time series data before the data analysis. The disparity condition was not run for 2 out of 15 participants in Experiment 1 because of technical issues. We acquired 4 scans per condition for each participant in Experiment 1, except 3/15 participants for whom we acquired only 3 scans for the contrast localizer condition. In Experiment 2, we acquired 4 scans of each condition per participant, except one for whom we only acquired 3 scans of each.

### Structural and functional MRI acquisition

Functional and structural MRI data were collected on a General Electric Discovery 750 (General Electric Healthcare) equipped with a 32-channel head coil (Nova Medical) at the Center for Cognitive and Neurobiological Imaging at Stanford University. For each participant, we acquired two whole-brain T1-weighted structural datasets (1.0 × 1.0 × 1.0 mm resolution, TE = 2.5 ms, TR = 6.6 ms, flip angle = 12, FOV = 256 × 256) that were used for tissue segmentation and registration with atlas-based ROIS and retinotopy template (see below). In both experiments, a multiplexed EPI sequence^80^ was used for the functional data, which allowed for the collection of 60 horizontal slices (2.0 × 2.0 × 2.0 mm resolution, TE = 30 ms, TR = 2000 ms, flip angle = 77, FOV = 220 × 220), resulting in whole-brain coverage.

### fMRI preprocessing and analysis

Preprocessing of anatomical and functional data was performed using fMRIPprep 1.0.8^81^ (RRID:SCR_016216), which is based on Nipype 1.0.0^82^ (RRID:SCR_002502). Each T1w (T1-weighted) volume was corrected for intensity non-uniformity (INU) using N4BiasFieldCorrection^83^ (ANTs 2.2.0). A T1w-reference map was computed after registration of the T1w images (after INU-correction) using mri_robust_template^84^ (FreeSurfer 6.0.1; RRID:SCR_001847). The T1w-reference was then skull-stripped using antsBrainExtraction.sh (ANTs 2.2.0), using OASIS30ANTs as target template. Brain surfaces were reconstructed using FreeSurfer’s recon-all^85^, and the brain mask estimated previously was refined with a custom variation of the method to reconcile ANTs-derived and FreeSurfer-derived segmentations of the cortical gray-matter implemented in Mindboggle^86^ (RRID:SCR_002438). Spatial normalization to the ICBM 152 Nonlinear Asymmetrical template version 2009c^87^ (RRID:SCR_008796) was performed through nonlinear registration with antsRegistration^88^ (ANTs 2.2.0, RRID:SCR_004757), using brain-extracted versions of both T1w volume and template. Brain tissue segmentation of cerebrospinal fluid (CSF), white-matter (WM) and gray-matter (GM) was performed on the brain-extracted T1w using fast^89^ (FSL 5.0.9, RRID:SCR_002823).

The following preprocessing was performed on the functional data. First, a reference volume and its skull-stripped version were generated using a custom methodology of fMRIPrep. A deformation field to correct for susceptibility distortions was estimated based on a field map that was co-registered to the BOLD reference, using custom fMRIPrep workflow derived from D. Greve’s epidewarp.fsl script and further improvements of HCP pipelines^90^. Based on the estimated susceptibility distortion, an unwarped BOLD reference was calculated for a more accurate co-registration with the anatomical reference. The BOLD reference was then co-registered to the T1w reference using flirt^91^ (FSL 5.0.9) with the boundary-based registration^92^ cost-function. Co-registration was configured with nine degrees of freedom to account for distortions remaining in the BOLD reference. Head-motion parameters with respect to the BOLD reference (transformation matrices, and six corresponding rotation and translation parameters) are estimated before any spatiotemporal filtering using mcflirt^93^ (FSL 5.0.9). The BOLD time-series were then slice time corrected the data using AFNI’s 3dTShift^94^ such that all slices were realigned in time to the middle of each TR and resampled into their original, native space by applying a single, composite transform that corrected for head-motion and susceptibility distortions. These resampled BOLD time-series were thus in registration with the T1w reference and will be referred to as preprocessed BOLD in original space, or just preprocessed BOLD. Many internal operations of fMRIPrep use Nilearn 0.4.0^95^ (RRID:SCR_001362), mostly within the functional processing workflow. For more details of the pipeline, see the section corresponding to workflows in fMRIPrep’s documentation.

We then used several AFNI^94^ functions to perform additional analysis steps: After eliminating dummy TRs, preprocessed BOLD time-series were scaled (each voxel was scaled to a mean of 100, and values were clipped at 200), and de-trended (removing components corresponding to the six head motion parameters, as well as Legendre polynomials of order 0 (constant signal), 1 (linear drift) and 2). Brain surfaces output by recon-all were converted to AFNI SUMA format using the @SUMA_Make_Spec_FS function. Finally, the 3dVol2Surf function was used to project the volume data onto each subject’s native surface as well as onto a standardized surface based on a icosahedron with 198812 nodes (linear depth of 141, see^96^). The native surface was used for ROI analysis, while the standardized surface was used for whole-brain analysis.

The remainder of the analysis was performed in custom Python code. The time-course data were first averaged across the scans for each condition, and then across the voxels within each visual region-of-interest (ROI). We then applied a Fourier transform to the average time-course for each ROI, omitted DC, multiplied the spectrum by 2 to get the single sided spectrum, and scaled by dividing with the number of samples in the time-course. We selected the complex value at the stimulus frequency (10 cycles per scan) for each participant, within each ROI, and used it for statistical analysis. We also used the average time-course to compute the mean cycle for each ROI. The order of the A and B block was mistakenly reversed for the disparity condition in Experiment 1, such that the B block was shown first. To facilitate easy comparison with the other conditions, we corrected for this by shifting the phase of the complex value by 180°, and adding 6 NaN-valued dummy TRs in the beginning of each run prior to computing the mean cycle. For the whole-brain analysis (see below), we performed the same Fourier analysis on a voxel-by-voxel basis, without averaging across ROIs, which gave us a complex value at the stimulus frequency, for every voxel in each participant.

### Visual regions-of-interest

Topographically organized visual ROIs were derived from a probabilistic atlas^97^. The atlas was generated by functionally defining 25 topographic ROIs covering 22 visual areas in ~50 individual participants, standardizing each participants’ surface using icosahedral tessellation and projection^96^, and then assessing the likelihood, across participants, of any particular vector on the standardized surface belonging to a particular ROI^97^. The atlas was defined using a maximum probability approach, which considers a given vector as part of the set of ROIs if it is more often found within the set, than outside the set, across participants. If this is the case, the vector is then assigned the value of the most likely ROI, and if not, it is considered to be outside the set of ROIs. The maximum probability approach captures much of the overall structure of ROIs defined for individual subjects and generalizes well to novel participants that did not contribute to the atlas generation^97^.

We downloaded the atlas from http://scholar.princeton.edu/sites/default/files/napl/files/probatlas_v4.zip and converted the ROIs from standardized surface space to native surface space for each of our participants, using nearest-neighbor interpolation. We removed vertices that were more than 1 edge away from the main cluster of each ROI, to ensure that all ROIs consisted exclusively of contiguous vertices. This step eliminated small speckles, while having minimal effect on the overall structure and extent of the ROIs. We excluded four ROIs from our analysis, IPS4 and 5, TO2 and FEF, because of their small size in the probabilistic atlas. We also excluded the dorsal and ventral segments of V1, V2 and V3, which were analyzed using the sub-ROI approach described below. This gave us a total of 15 bilateral ROIs to analyze.

To derive an independent estimate of the response in regions of early visual cortex responding to different eccentricities in the visual field, we used a template developed by Benson and colleagues^52^ that accurately predicts the location and retinotopic organization of early visual areas V1-V3, using only the cortical anatomy. After transforming the template data to match the specific cortical topology of each participant, we could now sub-divide the ROIs in early visual cortex, for each participant, by selecting voxels within the dorsal and ventral segments of V1, V2 and V3, defined based on the probabilistic atlas, that were responsive to a given range of eccentricities. We generated 24 overlapping sub-ROIs for each early visual area, centered on radii ranging from 0.25° to 6.0°, separated by 0.25°, and each spanning 0.5°/vis. angle.

For both ROI analyses, we excluded any participant for whom the combined number of vertices across both hemispheres was less than 50, and any ROI where less than 3 participants reached that criterion was excluded from analysis. This cut-off meant that for V2, the sub-ROI centered at 0.25° was not included in the analysis, while for V3, sub-ROIs centered at 0.25 and 0.5° were not included. For most of the remaining sub-ROIs (and all ROIs outside early visual cortex), all participants survived the criterion and were included. At least 10 participants survived per sub-ROI, except for the sub-ROI centered at 0.25° in V1, which in Experiment 2 only included 8 participants.

### Vector-based statistics

We computed the average phase and amplitude at the stimulus frequency using a vector-based approach, in which the real and imaginary part of the complex value was averaged separately across participants, and then combined so that vector-average amplitude and phase could be computed. The real and imaginary values used to compute the vector-average amplitudes in Experiments 1 and 2 have been plotted for the most significant negatively and positively signed V3 sub-ROIs, as well as for V3B, in Supplementary Figs 1 and 2. Error bars were computed using a geometrical approach, in which a two-dimensional error ellipse is computed, which describes the standard error of the mean response amplitude. The upper and lower error bounds were computed as the longest and shortest vectors from the origin to the error ellipse (a detailed describtion of this approach can be found in^98^). Statistical tests for significance of responses in individual ROIs were run as one-sample Hotelling’s *t*^2^ tests of the null hypothesis that the two-dimensional data set containing the real and imaginary parts of the complex value at the stimulus frequency was equal to [0,0]^99^. Note that this vector-based approach means that both amplitude and phase, and their consistency across participants, contributes to our reported estimates of mean amplitude, error and statistical significance. Following this logic, we used a two-sample Hotelling’s *t*^2^ to compare responses across the two experiments. Both one-sample and two-sample tests were run using the ICSNP^100^ package in R^101^.

We multiplied mean amplitude with phase sign, to illustrate the phase of the responses. We computed the phase sign in the following way. First, response phase was converted to response delay in seconds following the onset of the A block, adding 1 second to the results to account for the fact that voxel time-series were shifted to the center of each 2-second TR during slice-timing correction. This response delay, indicating the timing of the peak response, was now used to derive the phase sign of the response. Response delays at no less than 2 seconds into a block and no more than 2 seconds into the next, were associated with that block (given positive signs for A blocks, negative signs for B blocks), under the logic that responses peaking during the first 2-sec TR of a block would more likely be associated with the preceding block. As an additional precaution against ambiguity in the signing of responses, ROIs that peaked within one TR of the crossover-point (i.e. during the first four seconds of a block) were given signs, but not considered significant, even if the one-sample test was significant at α = 0.05. The goal was to ensure that positively and negatively signed ROIs that were considered significant peaked at least 2 TRs apart. In Experiment 1, this procedure affected only LO-2 in the contrast condition, and none of the sub-ROIs. In the disparity condition of Experiment 2, seven V1 sub-ROIs were affected (centered on: 2.5°, 2.75°, 3°, 3.25°, 3.5°, 3.75° and 4°) and one V2 sub-ROI (centered on: 2.75°). The procedure affected no other ROIs or sub-ROIs that otherwise reached significance.

### Whole-brain analysis

To provide an overview of the effect of our conditions across the whole brain, and account for any potential effects outside our set of visual ROIs, we mapped the complex values at each voxel onto a standardized cortical surface based on a icosahedron with 198812 nodes (linear depth of 141, see^96^), for each participant. This surface-based normalization across participants offers several advantages over volume-based approaches to group analysis^102^, most importantly by considering the structure of cortical sulci and gyri, as opposed to Talairach registration and other types cross-subject normalization in volume-space, which is likely to blur activations across neighboring banks of a sulcus^103^. We then applied the cross-subject analysis that we used for the ROIs and sub-ROIs, at each node of the standardized surface, using the real and imaginary values to compute the vector-average amplitude and response delay, and run the one-sample Hotelling’s *t*^2^ test for significance. We then plotted the response delay in seconds for each node on the surface, and thresholded the data at α = 0.05 based on the Hotelling’s test. We also applied the same restriction against ambiguous responses as described for the ROIs, and did not plot nodes that peaked during the first four seconds of a block.

## Supplementary information

Supplementary Figures

## Data Availability

The raw data from Experiments 1 and 2 are available at http://openneuro.org/datasets/ds001984 and http://openneuro.org/datasets/ds001978, respectively.
