# Supplementary material for: Image Segmentation Based on Relative Motion and Relative Disparity Cues in Topographically Organized Areas of Human Visual Cortex
Source: Sci Rep. 2019 Jun 26;9:9308. doi: 10.1038/s41598-019-45036-y (PMC6594975; doi:10.1038/s41598-019-45036-y)
Supplement: Supplementary file 1 — Supplementary Figures [file 41598_2019_45036_MOESM1_ESM.pdf]

Image segmentation based on relative motion and relative disparity cues in topographically organized areas of human visual cortex

Supplementary Figures

Peter J. Kohler*[a], Benoit R. Cottereau[b,c] and Anthony M. Norcia[a]

[a]Department of Psychology, Stanford University

Jordan Hall, Building 420, 450 Serra Mall, Stanford, CA 94305

[b] Université de Toulouse, Centre de Recherche Cerveau et Cognition, Toulouse, France

[c] Centre National de la Recherche Scientifique, Toulouse Cedex, France

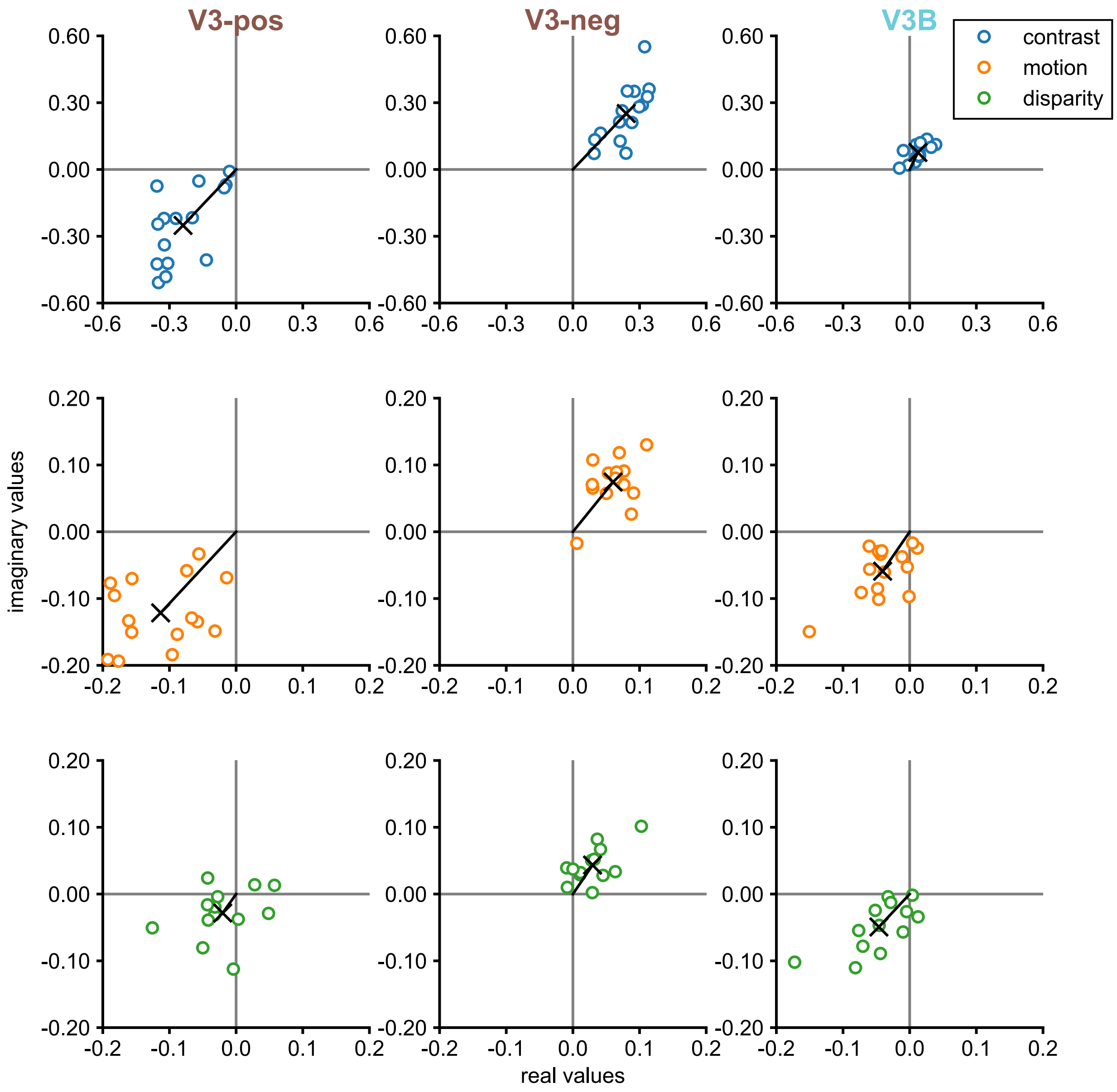

Supplementary Figure 1: Real and imaginary parts of the response in three example ROIs from Experiment 1: Each dot indicates the real and imaginary values from an individual subject (n=15 for contrast and motion, n = 13 for disparity). The black "x" indicates the vector average. The first two columns show values from the positively and negatively signed V3 sub-ROIs ("V3-pos" and "V3-neg") that are highlighted with up and down arrows, respectively, in Figure 2. For comparison, the real and imaginary values from area V3B are plotted in the rightmost column.

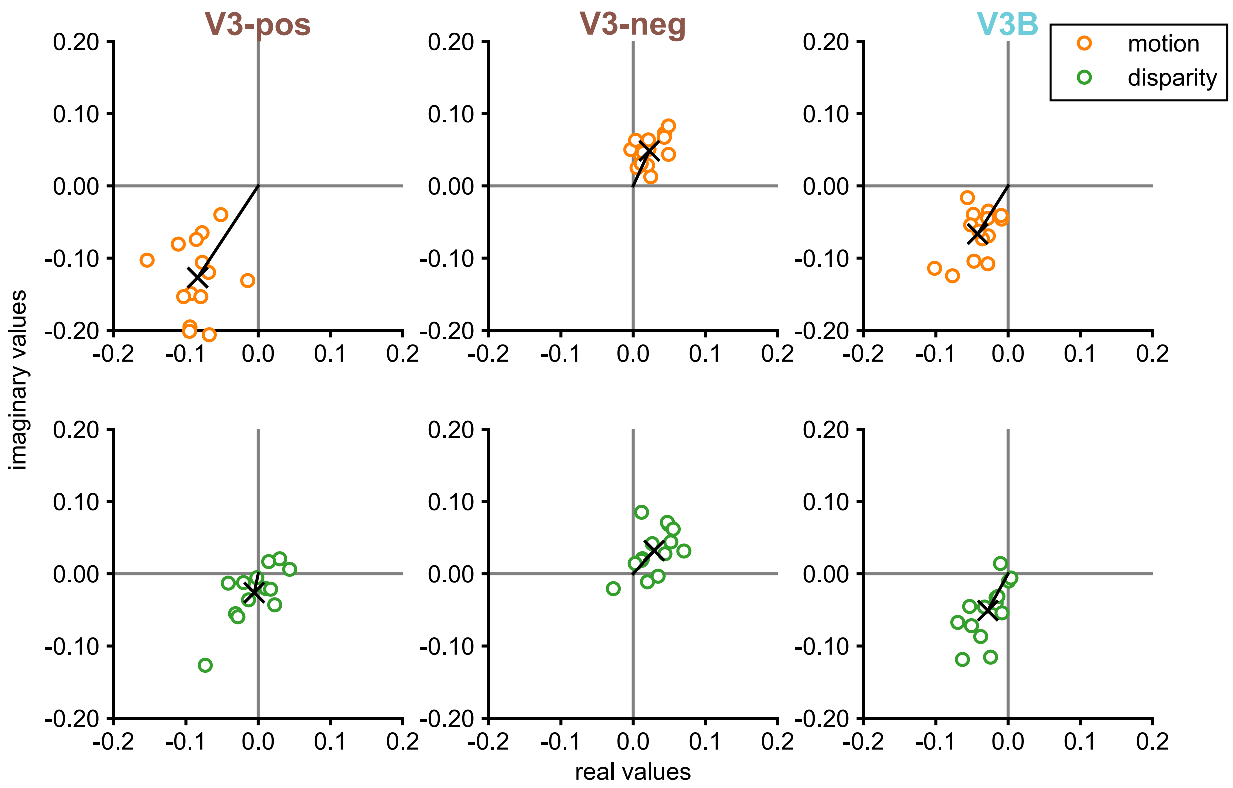

Supplementary Figure 2: Real and imaginary parts of the response in three example ROIs from Experiment 2: Each dot indicates the real and imaginary values from an individual subject (n=14). The black "x" indicates the vector average. The first two columns show values from the positively and negatively signed V3 sub-ROIs ("V3-pos" and "V3-neg") that are highlighted with up and down arrows, respectively, in Figure 4. For comparison, the real and imaginary values from area V3B are plotted in the rightmost column.
